# Evaluating Disparities in Elderly Community Care Resources: Using a Geographic Accessibility and Inequality Index

**DOI:** 10.3390/ijerph15071353

**Published:** 2018-06-27

**Authors:** Hui-Ching Wu, Ming-Hseng Tseng

**Affiliations:** 1 Department of Medical Sociology and Social Work, Chung Shan Medical University, Taichung 402, Taiwan; 2 Social Service Section, Chung Shan Medical University Hospital, Taichung 402, Taiwan; 3 Department of Medical Informatics, Chung Shan Medical University, Taichung 402, Taiwan; mht@csmu.edu.tw

**Keywords:** geographic accessibility, spatial inequality, resource allocation, community care, social support, elderly health, aging in place

## Abstract

This study evaluated geographic accessibility and utilized assessment indices to investigate disparities in elderly community care resource distribution. The data were derived from Taiwanese governmental data in 2017, including 3,148,283 elderly individuals (age 65+), 7681 villages, and 1941 community care centers. To identify disparities in geographic accessibility, we compared the efficacy of six measurements and proposed a composite index to identify levels of resource inequality from the Gini coefficient and “median-mean” skewness. Low village-level correlation (0.038) indicated inconsistencies between the demand populations and community care center distribution. Method M6 (calculated accessibility of nearest distance-decay accounting for population of villages, supplier loading, and elderly walkability) was identified as the most comprehensive disparity measurement. Community care policy assessment requires a comprehensive and weighted calculation process, including the elderly walkability distance-decay factor, demand population, and supplier loading. Three steps were suggested for elderly policy planning and improvement in future.

## 1. Introduction

The World Health Organization’s policy framework for “active aging” and “aging in place” defined active aging as “the process of optimizing opportunities for health, participation and security to enhance quality of life as people age” [1]. Among the determinants of active aging, those related to social environment have great potential to influence policy frameworks. Therefore, “aging in place” can be implemented through policies promoting community participation and neighborhood engagement among the elderly.

Because of decreased mobility, the health of the elderly may be more influenced by their neighborhoods. Thus, community participation is a key factor in the wider context of social determinants of elderly health. Community-based social support networks can reduce social isolation, provide emotional support, and increase independence among the elderly, thus enhancing well-being and promoting better overall quality of life outcomes [2].

Successful aging is multidimensional, encompassing physical, functional, psychological, and social aspects of health [3]. Thus, promotion of the quality of life among the elderly requires opportunities to participate in society, as well as medical care services. An important factor influencing social participation by the elderly is the geographic accessibility of community care resources. Accessible social activities link the elderly with social support networks, thereby enhancing mental health and overall well-being [4,5,6,7]. Spatial factors, particularly interactions between walkability, population density, and social cohesion strongly affect the elderly’s ability to establish and maintain social networks. Therefore, improving aging in place through the elderly’s ability to participate in community life requires strategies that consider how the neighborhood and individual factors interact [4]. 

Although a number of studies have examined the geographic accessibility of the elderly to community care resources, these have largely focused on the quantity of resources, and limited research only has employed measurements to assess disparities in resource distribution. To address this limitation in traditional resource-assessment methods, this study considered multiple factors based on the concept of “aging in place”, including the demand population, community care suppliers’ capacity, suppliers’ loading, the nearest distance to community care suppliers, and distance-decay effects. The methodologies and the findings in the present study demonstrate applications of community care resources allocation assessment that can inform policy-making, planning, and improvements in long-term care in Taiwan and other countries.

### 1.1. Community Care Resources and Active Aging

The equal allocation of community care resources is a major factor in the elderly’s ability to engage in society, empower their communities, and improve overall health and well-being [8]. Elements of successful aging include the maintenance of health and cognitive function, the reduction of disease and disability, ongoing social participation, and interpersonal and productive activities [9]. Participating in social activities can delay and avoid physical, psychological, and social aging and, then, promote successful aging. 

A functional community care organization provides health promotion activities and social support systems. Community care organizations promote elderly participation in social activities and interpersonal communication, thus enhancing life satisfaction as derived from their social support networks [10]. 

### 1.2. Accessibility Assessment of Community Care Resources

Encouraging the elderly to participate in community activities fosters active aging [11]; however, often, transportation restricts movement among the elderly [12]. Community-based elderly care resources can reduce such difficulties, thus enhancing engagement through the settings and institutions, norms, and trust deriving from participation in social networks, and systemic efforts toward social cohesion and collective action [13]. Accessible community care resources enhance elderly participation in the social networks that provide support that effectively promotes well-being. 

Geographic accessibility assessments have been applied widely in the health-care field [14,15,16,17,18,19,20,21], elderly learning resources [22]. Most studies of resource accessibility have focused on the distribution and equality of medical resources, measuring various relevant aspects of access, whereby system and population descriptors are process indicators, and utilization and satisfaction comprise major outcome indicators [23]. Studies measuring the geographic accessibility of elderly community care resources are rare; yet, this is an important policy issue. For this reason, we investigated the distribution of elderly community care resources to assess inequalities in geographical accessibility and resources allocation with the aim of developing policy recommendations regarding the distribution of long-term care resources.

In this study, we investigated three issues:

- The geographic accessibility of elderly community care resources.
- Inequalities in the accessibility of elderly community care resources.
- A multi-factorial method to measure geographic accessibility to community care resource allocation.

Our study compared six methods to calculate geographic accessibility, employed correlation coefficients to assess the relationship of the demand side (the demand of the population of villages) and the supply side (the amount of community care centers) and estimated the Gini coefficient to compare the inequalities between counties. Although this study focuses on the care resources allocation of the Taiwanese elderly community, its methodologies and results could also provide policy assessment implications for other countries.

## 2. Materials and Methods

### 2.1. Data Collection: Study Area and Datasets

This study considered three factors in measuring geographic accessibility of the elderly community care resources: supplier, demand population, and the geographic relationship between supplier and demand population. The study data was obtained from the Taiwanese governmental open data. On the supply side, the suppliers’ capacity and addresses were identified from community care centers based on the Ministry of Health and Welfare (Taipei, Taiwan) [24]. On the demand side, the distributions of demand populations were estimated from the Ministry of Internal Affairs NGIS Social and Economic Information Service [25]. Village-level road network distances were computed based on the data of the Ministry of Transportation and Communications [26].

Neighborhood was primarily operationalized using census-defined boundaries. Recent studies on neighborhood and health for the elderly were identified [27]. According to the Taiwanese government’s long-term care policy (“Long-Term Care 2.0”) [28], the service population is defined as individuals 65 years and older, and the total solution of community care framework is based on the village-level for the goal of “aging in place.” Thus, in 2017, the demand population for this study comprised 3,148,283 individuals. The study area covered 19 counties, 349 townships, 7681 villages, and 1941 community care centers in Taiwan (Main Island).

### 2.2. Measuring Geographic Accessibility to Elderly Community Care Resources

A widely used criterion for access to facilities classifies access based on geographic factors, thus emphasizing the spatial separation between supply and demand as a barrier or a facilitator, or non-geographic factors, which stress non-spatial barriers or facilitators [29]. This study measures the geographic accessibility of elderly community care centers in Taiwan to investigate the equality of long-term care resources distribution.

Common measurements of geographic accessibility include travel time or distance between the demand population’s location and the facility and the number of suppliers in each administrative district [30,31,32]. Using travel time to the nearest supplier to assess geographic accessibility may relate to the number of the service suppliers [33]. Thus, using travel time to evaluate accessibility is not always a determining factor, since other factors such as facility capacity, insurance acceptance, and travel costs can affect access [34]. 

It is a complicated task to account for disparities in spatial relationships between the supply and demand sides. Thus, in this study, six accessibility measurements were evaluated to estimate levels of geographic accessibility from 7681 villages and 1941 elderly community care centers in Taiwan in 2017, taking into account both accounting factors of distance and the amount of community care centers, as well as supplier’ points (supplier loading) (Table 1). The ESRI ArcGIS 10.5.1 (Esri, Redlands, CA, USA) “Model Builder” tool incorporated with the “Network Analyst extension” module and SQL programming from Microsoft SQL Server 2014 (Microsoft, Redmond, WA, USA) were used to calculate the accessibility measures.

The main goal of the Taiwanese government’s long-term care policy [28] is to achieve “aging in place”, and its primary mission is to establish a good-quality neighborhood and a community-based accessible, affordable, universal, long-term care service system. To create a comprehensive care system that integrates medical care, long-term care services, housing, prevention, and social supports for the elderly within a 30-min drive, the system (“Long-Term Care 2.0,” 2017) includes “Tier A–Community integrated service centers”, “Tier B–Combined service centers”, and “Tier C–LTC stations around the blocks.” The basic executive unit (“Tier C–LTC stations around the blocks”) relies on village-level community care centers to satisfy the elderly’s accessibility. Restricted by governmental funding policy, the elderly can choose one community care center only within their census-registered county, and most choose the one with the shortest distance. Thus, all six methods estimate accessibility based on the assumption of the nearest road distance in this study.

*Mx_i_* (i.e., *M1_i_*, *M3_i_*, *M4_i_*, *M5_i_*, and *M6_i_*) is the score of geographic accessibility at the centroid of village *i* using method M*x*. *M2_j_* is the score of supplier loading at the elderly community care center *j* using method M2. *S_j_* is the capacity of the elderly community care center *j*. *S_i_* is the capacity of the elderly community care centers at the centroid of village *i*. *P_i_* is the demand population over 65 years in village *i*, and *d_ij_* is the road network travel distance between village *i* and the elderly community care center *j*.

Method M1 employed road network distance [31] to measure the impact of travel time on geographic accessibility and estimated the within-jurisdiction road network travel distance from the centroid of each village to the nearest elderly community care center within every jurisdiction (i.e., county). Method M1 shows the real traffic distance between the elderly at every village and the nearest center within every county.

Method M2 estimated the nearest distance supplier loading within every county, whereby a larger score denoted lower geographic accessibility. For example, if the community care center is the common nearest supplier to several within-jurisdiction villages, summing up the total demand population at those nearby villages obtains the nearest supplier loading. 

Method M3 measured the supplier–population ratio of the elderly community care centers within an “official region”, defined by village level in this study. As an easy and simple method to calculate the ratio, often, method M3 is used as a basic indicator to evaluate resource shortage areas; however, it has two disadvantages for evaluating geographic accessibility: it cannot explain the related spatial variations within a specific area, and it assumes that the demand population would not seek resources across the region’s borders [35]. In other words, method M3 assumes that the elderly in a specific area have equal opportunity to acquire resources regardless of the travel distance.

A comprehensive accessibility measure should include possible influencing factors for the elderly’s access to these community care resources, and methods M4–M6 simultaneously account for the demand population of villages, supplier capacity, supplier loading, and distance. Methods M4–M6 consider the different distance-decay function to estimate the impact of different distance scales (Table 1), since the elderly’s physical activity can be restricted by these. For each factors’ weights are not known, and there are no surveys that have been done to estimate the importance of these factors. We applied “equal weighting” method to minimize maximum possible disagreement over all possible distributions [36].

Methods M4 and M5 assume that the nearest community care center is always accessible whatever the nearest distance scale for the elderly within each county—that is, the elderly in each village can access the nearest resources; however, this neglects the travel distance-decay factor in elderly walkability. Method M4 calculated the nearest distance-decay effect by considering within-jurisdiction accessible resources and the nearest road distances to estimate accessibility. Method M5 estimated accessibility by additionally considering the supplier’s loading, such that the elderly population of villages near the common elderly community center were summed up and were considered as sharing that center’s resources. Thus, method M5 can enhance the assessment of the adequacy of resource allocation.

Walkability is a measure of how friendly an area is to walking. Often, the elderly are disadvantaged in terms of mobility; thus, method M6 is based on the same concept as method M5, but also accounts for elderly walkability. The walkable accessibility of community-based care resources can improve the elderly’s health and aging in place. Although walking seems like a simple activity, it is actually a complex symphony involving many of the body’s systems, whereby our bodies must coordinate balance, muscle contraction, and relaxation, as well as adjust the cardiovascular system. Therefore, a friendly, community-based elderly care center should consider the elderly’s neighborhood walkability and physical abilities [27,37]. The appropriate walking speed for older pedestrians is 0.91 m/s [38]. Method M6 accounted for village population and supplier loading in calculating the accessibility of the nearest distance-decay. Method M6 presents a more realistic measure of accessibility, because it considers the elderly’s walkability and care in village-level neighborhood. Method M6 set 3 km as the appropriate nearest supplier’s distance. Considering that the elderly may decrease walking speed to rest, method M6 estimated about 60-min of walking time, and the nearest supplier’s road distance to the elderly was determined to be within 3 km. The distance-decay weight was set to one when the distance is within 3 km, and inversely varies the nearest distance after 3 km. 

An extension to method M5, method M6 incorporated the elderly’s walkable distance, supplier loading, and the demand population in each village. Thus, the within-jurisdiction accessibility measure using method M6 provides the most comprehensive assessment of community care resources.

### 2.3. Domain Partition OD Cost Matrix Calculation Approach

In network-based geographical accessibility analysis, setting the searching distance is an important parameter in an origin-destination (OD) cost matrix calculation approach. Walsh, Cullinan, and Flannery [31] examined the full study domain to consider all distance weights in their study; however, Cabrera-Barona, Blaschke, and Gaona [32] used the maximum distance threshold of 1.2 km to identify supply services.

Under the existing public administrative system in Taiwan, the allocation of public service budgets is governed by county-level administrative units (such as the county-level governments), which often determines the service scope of resources, and qualified users of elderly community care resources are limited to the census-registered residents of each county. Therefore, the county-level administrative district is a suitable assessment unit with which to estimate the accessibility of community care resources in Taiwan. This study designated the administrative district as the county (or city), and each district has its own resource-searching distance.

**Figure 1:**
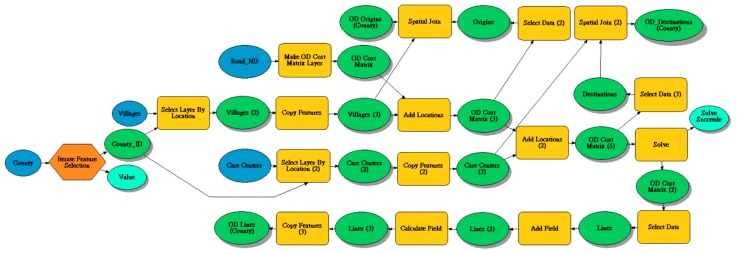
Domain partition OD (origin-destination) cost matrix calculation approach using the “Model Builder” programming of the ESRI ArcGIS.

This study developed a domain partition OD cost matrix calculation approach (Figure 1). First, we used the ESRI ArcGIS “Model Builder” (Esri, Redlands, CA, USA) programming to implement the domain partition algorithm for dividing the whole study area (Taiwan Main Island) into nineteen domains into county-level administrative districts to select within-jurisdiction feasible solutions. Then, we applied the ArcGIS “Network Analyst extension” to complete an origin–destination cost matrix calculation for each county. The within-jurisdiction feasible solution means that the accessibility calculation must meet practical policy restrictions for utilizing resources. That is, the elderly need to be residents registered in that specific region. Finally, we used Microsoft SQL Server 2014 to calculate the six geographic accessibility measurements.

### 2.4. Spatial Inequality Index of Elderly Community Care Resources

In addition to the accessibility of elderly community care resources, this study examined inequalities in elderly community care resource accessibility between counties by applying the Gini coefficient to compare geographic accessibility between administrative districts.

The Gini coefficient is a measure of statistical distribution that was first proposed as a measure of income or wealth inequality [39] by showing the income distribution of a country’s population, and, often, it is applied to interpret relative deprivation in a society [22,40]. It measures inequality among values of a frequency distribution, whereby zero expresses perfect equality and one indicates maximal inequality. 

Interpretation of the Gini coefficient is controversial, because it is a relative measure index. Its main disadvantage is its inability to explain different distributions with the same Gini coefficient. Therefore, we further calculated the scores of “median-mean” and “max-min” to show the range of distributions and disparities in each county.

To investigate the actual distribution in each administrative district, we used data drill-down techniques to deepen and, then, mine into the content of the datasets, accessing information by starting with a general category (the county-level Gini coefficient values) and moving through the hierarchy to calculate down to the village-level Gini coefficient distribution.

## 3. Results

### 3.1. Resource Allocation Correlation of Demand Population and Community Care Centers

The Taiwanese government’s long-term care policy (“Long-Term Care 2.0”, 2017), defines the service population as individuals aged 65 years and older, and the total solution of community care framework is based on the village-level. To assess whether the community care centers’ allocation satisfies the demand population’s need, we calculated the population-to-centers correlation coefficient. The population-to-centers correlation coefficients display different meanings in different levels of administrative districts, whereby the coefficient is 0.038 at the village-level, 0.362 at the town-level, and 0.410 at the county-level (Table 2). This indicates that the low village-level correlation coefficient means the community care center’s allocation is inconsistent with the demand population’s location and, thus, deviates from the policy goal.

**Figure 2:**
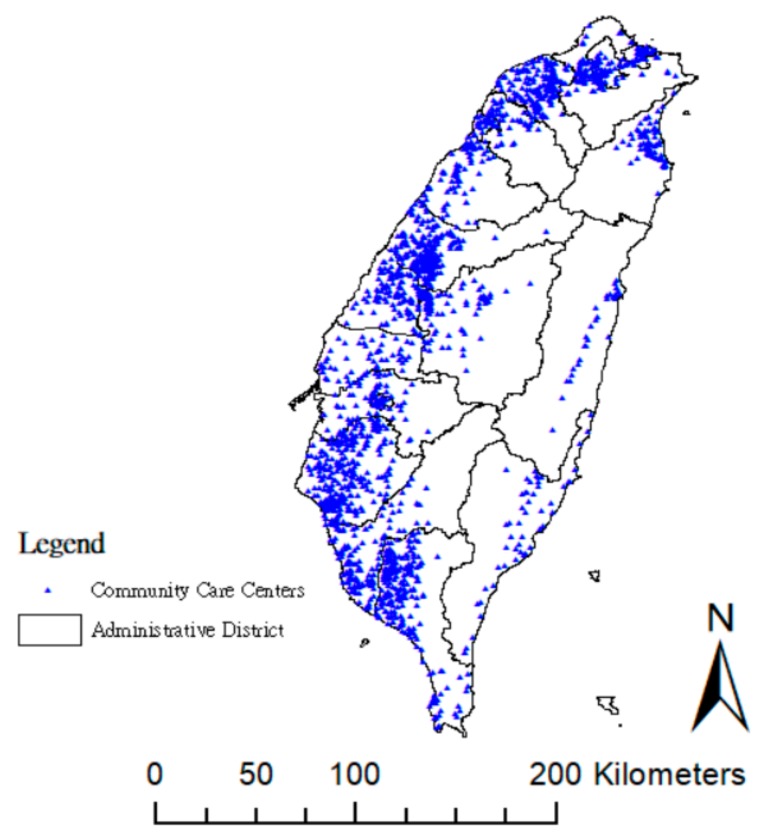
Geographic distribution of community care centers in Taiwan, 2017.

Figure 2 shows the distribution of the community care centers, whereby the blue dots on the western side of Taiwan present high density, while those on the eastern side are more dispersed, thus indicating less accessibility.

### 3.2. Geographic Accessibility of Elderly Community Care Resources

Method M1 estimated the geographic accessibility based on distance. As Table 3 shows, the “max-min” score is 39.355 km and SD (standard deviation) is 2.302, which indicates a large disparity in resource distances, most notably between rural and urban counties, whereby the nearest road distances of rural counties’ community care centers are larger than those of urban areas.

Method M2 compared the nearest distance supplier loading to identify geographic accessibility (Table 3), whereby a zero min. score indicates that some centers may compete with others for demand population, because their locations are too close; therefore, they may encounter the crisis of lacking demand population. Among the max scores, some suppliers estimated providing services for a demand population of only a few thousand, while others have demand populations of up to nearly 40,000 people, which indicates that some community care centers may be overloaded. If some centers are the nearest supplier for the surrounding villages, these demand populations would all share the same supplier.

Method M3 provided the simplest assessment by showing rough estimates of the locations of the elderly in relation to the nearest community care center within every village, and the resulting accessibility scores are smaller than those achieved from other methods, as shown in Table 3. These scores (mean = 0.859, median = 0.000, min = 0.000, and max = 36.364) were summed up from village-level to county-level administrative districts and indicate very low accessibility. Method M3 supposes that the demand population would not seek community care centers across their census-registered villages’ borders. To address this limitation, we proposed methods M4–M6 to enhance the measurement of geographic accessibility.

Methods M4–M6 were all calculated on the basis of network distances, and the SD scores of methods M5 (1.244) and M6 (1.265) are smaller than that of method M4 (4.474), which indicates that methods M5 and M6 reduce variation more than method M4, such that the intervals between minimum and maximum estimated by method M5 (23.228) and M6 (24.108) are smaller than those for method M4 (124.671). These results are because method M4 uses supplier capacity, but method M5 considers the supplier loading.

The scores of “median-mean” and “max-min” estimated by method M6 are larger than those by method M5. Because method M6 also calculated accessibility by nearest distance decay (3 km), it more clearly presents the accessibility difference or inequality between urban and rural areas. Then, the mean (0.811) and median (0.446) scores from method M6 show that 50% of the demand population’s accessibility is lower than average, thus indicating the inequality of community care centers’ accessibility when comprehensively accounting for walkability, the demand population, and supplier loading, thus indicating inequalities in community care center accessibility when comprehensively accounting for walkability, the demand population, and supplier loading. These scores suggest that most of the elderly will suffer poor community care accessibility, thus negatively affecting their social participation.

Table 4 shows the results of our comparison of the nearest road distances of community care centers between administrative districts using method M1. There are eight administrative districts for which the mean and SD scores are higher than average; their max scores are also larger than those of other districts. These eight administrative districts are all located in rural areas, and even in districts with larger numbers of community care centers, the nearest distances are larger than average. This indicates room for the improvement of accessibility. Based on the identification of districts with a dispersed distribution of community care centers as provided by method M1, policy-makers can support these centers (or add more centers) to increase the accessibility of community care centers for the elderly. 

In Table 5, all the median scores for community care center loading measures by method M2 are smaller than the means, and 10 districts have higher than average scores for mean and median, which suggests that supplier loading in these areas is at risk of overload.

Methods M4–M6 also computed and compared the quintile accessibility of community care resources (see Table 6) to determine the distribution of accessibility by identifying how many elderly people experience poor accessibility to the nearest elderly community care center. Obviously, all of the scores for method M4 are larger than those for methods M5 and M6, since the latter methods account for supplier loading along with supplier capacity. 

The medians measured by all six methods were lower than the means in Table 3, such that the accessibility of elderly community care centers is skewed to the right. Since the accessibility of some villages is extremely high or low, using the mean scores would not display the real accessibility of 50% of the village demand population to community care centers. Therefore, the median scores may be a more appropriate value than the mean to indicate real accessibility distribution, as they can further demonstrate issues of accessibility inequality between administrative districts. 

Table 7 shows the results of the estimated scores used to calculate the number of villages as categorized into different levels by dividing the scores of accessibility into quintiles and calculating the amount of villages, whereby scores of village-level accessibility below Q20 were categorized as “very low”, scores between Q20 and Q40 were categorized as “low”, scores from “Q40–Q60” were categorized as “fair”, scores from “Q60–Q80” were categorized as “high”, and scores above Q80 categorized as “very high.” 

**Figure 3:**
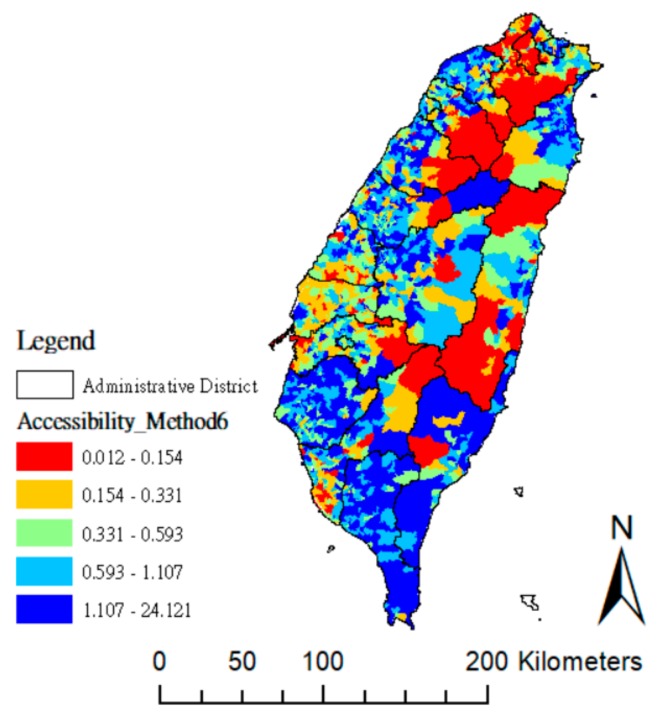
Quintile of village distribution as estimated by median of accessibility based on method M6.

As Figure 3 shows, the distribution is obviously unequal when the accessibility scores (measured by method M6) are grouped into quintiles. “High” and “very high” accessibility (light and dark blue areas) are mainly concentrated in the western, middle, and southern counties, while “low” and “very low” accessibility (red and orange areas) are concentrated in the north, central, and southern counties. These results can provide clear evidence regarding the amount and locations of “low” and “very low” villages, thus helping policy-makers to set their priorities in improving poor geographic accessibility.

### 3.3. Spatial Inequalities in Elderly Community Care Resources

Although the supplier capacity of elderly community care centers has increased every year, our study shows that this has only partly improved geographic accessibility, since some villages have not provided a community care center for the elderly yet. Therefore, the issue of geographic inequalities in accessibility is a major factor in assessing which regions have poor accessibility, pressure of large demand population, and unequal resource distributions. 

Table 8 presents the “median-mean” scores to show the skewness. In a perfectly symmetrical distribution, the means and the medians would be the same; however, if the mean is less than the median, the shape distribution is skewed to the right, while a positive skew means that the right tail is longer, such that the mass of the distribution is concentrated on the left. Therefore, the smaller the “median-mean” scores, the more average the distribution is. The “max-min” scores present the full range of extremes (minimum versus maximum), which helps to identify extreme disparities in resource allocation.

It can be seen that all of the “median-mean” scores are positively skewed, whereby the right tails are longer, and the mass of the distribution (“median”) is concentrated on the left. This “right-skewed” trend means that the median is smaller than the mean; that is, the accessibility of 50% of elderly people to community care centers is lower than the mean score. The Gini coefficients of methods M4–M6 are 0.497, 0.558, and 0.562, respectively, thus, indicating extremely unequal accessibility to community care resources.

Comparing the mean scores obtained from methods M4–M6 (see Table 9), all the scores of accessibility show a decreasing trend, which indicates a need for improved accessibility when simultaneously considering the factors of distance-decay, demand population, and supplier capacity and loading. The SD scores obtained from methods M4–M6 also show a decreasing trend, such that the variation estimated by methods M5 and M6 is smaller than method M4. Comparing the medians of methods M5 and M6 between different districts (Table 9), 13 districts show decreasing accessibility (marked with “*”). Applying method M6 to consider the effect of distance-decay threshold (3 km), the median scores present lower accessibility for the demand population of elderly in rural administrative districts.

Table 10 shows the estimated “median-mean” scores and Gini coefficients to display the inequality of resource allocation between administrative districts by methods M4–M6. The “median-mean” scores show the interval of distribution and skewness of inequality, and cases in which “median-mean” is smaller than average are marked with asterisks to indicate the unequal accessibility. The Gini coefficients are divided into two levels, such that 0.4 to 0.6 is categorized as “median inequality” and above 0.6 is categorized as “high inequality.” The composite index of inequality summed up the “*” of “median-mean” and Gini coefficient, such that level 1 indicates “low inequality” (“*”), level 2 represents “middle inequality” (“**”), and level 3 denotes “serious inequality” (“***”).

Comparing the Gini coefficients by methods M5–M6, all the administrative districts show unequal resource distribution. Our study proposed a “composite index of inequality”, categorized one district as having “serious inequality” (Taitung county), four districts with “middle inequality” (Kaohsiung City, Tainan City, Nantou County, and Pingtung County), and 14 districts with “low inequality.” An explanation for the “serious inequality” in Taitung County is its location in the east of Taiwan, where factors of distance decay, suppliers’ capacity, and loading interact closely. Among districts categorized as “middle inequality”, unequal accessibility in municipalities with larger administrative scales such as Kaohsiung City and Tainan City is attributable to higher demand populations, low supplier capacities, and far distances to the nearest community care center. Nantou County and Pingtung County are secondary administrative districts, where the public budgets are relatively smaller than those for municipalities, thereby causing unequal resource allocation. 

## 4. Discussion

Neighborhood social networks are important for enhancing the health and well-being of the elderly, and community-based care is closely associated with their participation in social activities [6,7]. Geographic accessibility is a significant factor in these social interactions; yet, previous research has rarely focused on the geographic accessibility of elderly community care resources. Thus, our study represents a significant contribution to the literature on elderly community care resource policy assessment. 

The results of our study have important implications for elderly community care policy. First, our comparison of six measurement methods of geographic accessibility and inequality indicated that the most comprehensive measurement is provided by method M6, which considers multiple factors (the nearest distance, the distance-decay accounting for elderly walkability, the demand population of villages, and the supplier loading); therefore, it can most realistically identify inequalities in geographic accessibility and resource allocation. The number of villages under Q40 (40.13%) for their demand populations’ geographic accessibility to community care resources should be improved (Table 7). The results showed that the medians of accessibility are lower than the means between administrative districts are (Table 9). Second, this study examined spatial inequality as evidenced by the Gini coefficient, median, and mean. Third, the different distance-decay measures indicate the impact of distance factors on the resource utilization. The distance-decay coefficient should consider elderly walkability and the policy goal of aging in place.

For future researches and policy-making decisions, three steps are suggested to apply method M6 for elderly policy planning:

Step 1. To describe the outline of community care resources allocation and identify which administrative district’s resources allocation should be improved, the correlation coefficient of demand population and supplier capacity can be calculated to assess the adequacy of resource allocation (Table 2). Moreover, the nearest road distance of community care resources by administrative districts can be computed to display the disparity of travel time (Table 4), and the supplier’s loading can be estimated to assess the appropriateness of the location (Table 5).
Step 2. Using method M6 to discriminate differences between administrative districts and estimate the spatial inequality of elderly community care resources, the “median-mean” and “max-min” scores can be calculated to display the deviance and the full range of extremes of geographic accessibility (Table 9), while the Gini coefficient can be estimated to show unequal levels, and the “composite index of inequality” can identify levels of inequality (Table 10). These scores can serve as a reference framework for the central government in setting its priorities for improving poor accessibility districts and planning more equitable public funding distribution policies.
Step 3. The improvement of local governmental budget distributions can be informed by dividing the number of villages into quintiles by scores of accessibility (Table 7), and community care resources allocation should prioritize those villages receiving scores below Q40 (“low” to “very low” accessibility). By simultaneously considering the nearest distance (Table 4) and supplier loading (Table 5), local governments can set a policy schedule to improve the geographic accessibility based on current accessibility levels.

## 5. Conclusions

This study combined sociological perspectives and a GIS-based approach called “domain partition OD cost matrix calculation” to examine the accessibility of elderly community-based care resources and to develop feasible within-jurisdiction solutions for policy support. Community-based care resources can be important social support systems in promoting elderly health. The results indicate that to promote equal resource allocation and reasonable geographic accessibility, assessing geographic accessibility should not only consider the amount of centers and travel distances, it should also require a comprehensive and weighted calculation process depending on distance-decay, demand population, and supplier loading. To construct a friendly, community-based care support system, it is recommended that future studies consider the impact of distance on elderly walkability. Although our study focuses on Taiwanese cases, the methodologies illustrate possibilities for future research to inform long-term care policy planning and implementation elsewhere.

Overall, these results remind us that policy assessments should not only estimate geographic accessibility but should also aim for more equitable resource distribution. The assessment of spatial resource allocation is a key issue for governmental agenda-setting, whereby geographic accessibility assessments should examine both the demand side (elderly demand population, rural/urban locations of administrative districts) and the supply side (distance-decay factor, capacity of community care centers). To optimize the spatial locations of community-based care resources, geographic accessibility and the equality of resource distribution need to be considered simultaneously. 

This study has some limitations. First, due to variability in the elderly’s transportation to community care centers, we calculated accessibility on the basis of road network distance, thus neglecting the availability of public transportation. A friendly public transportation system is an important factor in elderly mobility, particularly in an aging, low-fertility society. Hence, future studies can include public transportation in considerations of travel time. Second, owing to a lack of official data about the number of users for each elderly community care center, this study set the supply size as 1000 per demand population.

## Figures and Tables

**Table 1:** Definition of spatial accessibility measures.

Method	Description (Unit)	Formula	Distance Decay Function
M1	Nearest road distance (km)	$M1_{i}=\underset{j}{\min}d_{ij}$	N.A.
M2	Nearest distance supplier loading (people)	$M2_{j}=\underset{i}{\sum }\frac{P_{i}}{S_{j}}$	N.A.
M3	Accessibility of ownership averaged by official regions (1000*capacity/people)	$M3_{i}=\frac{S_{i}}{P_{i}}$	N.A.
M4	Accessibility of nearest distance-decay accounting for population of villages and supplier capacity (1000*capacity/people)	$M4_{i}=\underset{j}{\sum }\frac{S_{j}*f\left(d_{ij}\right)}{P_{i}}$	$f\left(d_{ij}\right)=\left\{\begin{array}{c} 1,\;\;d_{ij}=\underset{j}{\min}d_{ij}\\ 0,\;\;d_{ij}>\underset{j}{\min}d_{ij} \end{array}\right\}$
M5	Accessibility of nearest distance-decay accounting for population of villages and supplier loading (1000*capacity/people)	$M5_{i}=\underset{j}{\sum }\frac{S_{j}*f\left(d_{ij}\right)}{\sum_{k}P_{k}*f\left(d_{jk}\right)}$	$\;f\left(d_{ij}\right)=\left\{\begin{array}{c} 1,\;\;d_{ij}=\underset{j}{\min}d_{ij}\\ 0,\;\;d_{ij}>\underset{j}{\min}d_{ij} \end{array}\right\}$
M6	Accessibility of nearest distance-decay accounting for population of villages, supplier loading, and elderly walkability (1000*capacity/people)	$M6_{i}=\underset{j}{\sum }\frac{S_{j}*f\left(d_{ij}\right)}{\sum_{k}P_{k}*f\left(d_{jk}\right)}$	$f\left(d_{ij}\right)=\left\{\begin{array}{c} 1,d_{ij}=\underset{j}{\min}d_{ij}\&\;d_{ij}\leq 3km\\ \frac{3}{d_{ij}},\;d_{ij}=\underset{j}{\min}d_{ij}\&\;d_{ij}>3km\\ 0,\;d_{ij}>\underset{j}{\min}d_{ij}\; \end{array}\right\}$

**Table 2:** Summary statistics of 65+ population and community care centers measures by administrative district.

Administrative District	65+ Population	65+ Population, (%)	Number of Centers	Number of Villages	Centers-to-Population, (%)	Centers-to-Villages, (%)	Correlation Coefficient (Population-to-Centers)
Yilan County	69,013	2.19	80	233	0.12	34.33	Country-level: 0.410 Town-level: 0.362 Village-level: 0.038
Hsinchu County	65,305	2.07	39	192	0.06	20.31	
Miaoli County	84,034	2.67	85	274	0.10	31.02	
Changhua County	185,907	5.91	112	589	0.06	19.02	
Nantou County	81,566	2.59	88	262	0.11	33.59	
Yunlin County	119,761	3.80	50	388	0.04	12.89	
Chiayi County	93,296	2.96	55	357	0.06	15.41	
Pingtung County	127,325	4.04	227	455	0.18	49.89	
Taitung County	32,837	1.04	52	140	0.16	37.14	
Hualien County	49,484	1.57	33	177	0.07	18.64	
Keelung City	53,550	1.70	65	157	0.12	41.40	
Hsinchu City	49,406	1.57	26	122	0.05	21.31	
Chiayi City	37,128	1.18	22	84	0.06	26.19	
Taipei City	428,648	13.62	41	456	0.01	8.99	
Kaohsiung City	383,659	12.19	138	891	0.04	15.49	
New Taipei City	483,602	15.36	95	1032	0.02	9.21	
Taichung City	310,710	9.87	255	625	0.08	40.80	
Tainan City	265,121	8.42	304	752	0.11	40.43	
Taoyuan City	227,931	7.24	174	495	0.08	35.15	
**Total**	**3,148,283**	**100**	**1941**	**7681**			

**Table 3:** Summary statistics of community care resources accessibility measures.

Method	Mean	Median	SD	Min	Max	Median-Mean	Max-Min
M1 Nearest road distance (km)							
	1.596	0.956	2.302	0.004	39.359	−0.640	39.355
M2 Nearest distance supplier loading (people)							
	1625	898	2499	0	35883	−727	35883
M3 Accessibility of ownership averaged by administrative districts (1000*capacity/people)							
	0.859	0	2.108	0	36.364	−0.859	36.364
M4 Accessibility of nearest simple distance-decay accounting for population of villages and supplier capacity (1000*capacity/people)							
	3.892	2.915	4.474	0.329	125.000	−0.977	124.671
M5 Accessibility of nearest simple distance-decay accounting for population of villages and supplier capacity (1000*capacity/people)							
	0.824	0.465	1.244	0.028	23.256	−0.359	23.228
M6 Accessibility of nearest moderate distance-decay accounting for population of villages and supplier capacity (1000*capacity/people)							
	0.811	0.446	1.265	0.013	24.121	−0.365	24.108

**Table 4:** Summary statistics of the nearest road distance (in kms) of community care centers as determined by method M1.

Administrative District	Mean	SD	Min	Max
Yilan County	2.237 *	4.273 *	0.046	36.648
Hsinchu County	2.827 *	3.282 *	0.118	25.672
Miaoli County	1.937 *	2.146	0.032	23.142
Changhua County	1.532	1.190	0.013	7.232
Nantou County	2.798 *	3.424 *	0.069	22.149
Yunlin County	2.229 *	1.581	0.069	14.620
Chiayi County	2.938 *	2.950 *	0.092	22.101
Pingtung County	1.290	1.537	0.064	10.814
Taitung County	2.735 *	2.662 *	0.052	15.941
Hualien County	4.884 *	5.897 *	0.068	27.150
Keelung City	0.544	0.657	0.038	4.426
Hsinchu City	0.796	0.562	0.037	3.453
Chiayi City	0.651	0.486	0.022	2.762
Taipei City	1.025	0.885	0.025	6.067
Kaohsiung City	1.387	2.332 *	0.051	39.359
New Taipei City	1.437	1.949	0.032	22.130
Taichung City	0.968	1.168	0.019	9.830
Tainan City	1.002	0.947	0.004	8.833
Taoyuan City	1.006	1.627	0.015	21.407
**Average**	**1.596**	**2.302**		

* higher than “Average.”; SD: standard deviation.

**Table 5:** Summary statistics of the community care centers’ loading measures (in people) as identified using method M2.

Administrative District	Number of Centers	Mean	Median	SD	Min	Max
Yilan County	80	863	794	637	0	3332
Hsinchu County	39	1674 *	1014 *	1621	0	8309
Miaoli County	85	989	783	773	0	3603
Changhua County	112	1660 *	1190 *	1492	0	7693
Nantou County	88	935	739	721	0	4351
Yunlin County	50	2395 *	2115 *	1597	334	6547
Chiayi County	55	1714 *	1259 *	1437	131	6948
Pingtung County	227	561	398	528	0	3065
Taitung County	52	631	426	849	0	5046
Hualien County	33	1500 *	1274 *	1399	0	6811
Keelung City	65	824	714	672	0	3188
Hsinchu City	26	1900 *	1413 *	1511	148	6273
Chiayi City	22	1688 *	1665 *	1038	176	3872
Taipei City	41	10,455 *	9672 *	7904 *	501	35,883
Kaohsiung City	138	2780 *	1536 *	3093 *	0	16,787
New Taipei City	95	5091 *	4034 *	3882 *	0	16,752
Taichung City	255	1224	1020 *	917	0	4557
Tainan City	304	872	681	749	0	4804
Taoyuan City	174	1310	969	1197	0	9087
**Average**	**102**	**1625**	**898**	**2500**		

* higher than “Average.”; SD: standard deviation.

**Table 6:** Quintile accessibility of community care resources as measured using methods M4–M6 (estimated by 1000*capacity/people).

Method	Q5	Q25	Q50	Q75	Q95
M4 Accessibility of nearest simple distance-decay accounting for population of villages and supplier capacity					
	1.064	1.898	2.915	4.566	9.346
M5 Accessibility of nearest simple distance-decay accounting for population of villages and supplier loading					
	0.078	0.208	0.465	0.951	2.688
M6 Accessibility of nearest moderate distance-decay accounting for population of villages and supplier loading					
	0.071	0.191	0.446	0.933	2.752

**Table 7:** Village distribution as estimated by scores of accessibility based on method M6 (estimated by 1000*capacity/people).

Administrative District	Median of Accessibility	Number of Villages					
		Very Low <Q20	Low Q20~Q40	Fair Q40~Q60	High Q60~Q80	Very High >Q80	Total
Yilan County	0.812	3	15	36	96	83	233
Hsinchu County	0.366	32	44	59	27	30	192
Miaoli County	0.796	7	32	66	76	93	274
Changhua County	0.429	43	177	196	86	87	589
Nantou County	0.727	7	32	66	76	81	262
Yunlin County	0.301	36	183	87	54	28	388
Chiayi County	0.396	42	95	103	68	49	357
Pingtung County	1.259	0	6	47	137	265	455
Taitung County	1.081	1	25	12	32	70	140
Hualien County	0.434	35	30	38	50	24	177
Keelung City	0.933	0	10	18	63	66	157
Hsinchu City	0.447	1	45	36	34	6	122
Chiayi City	0.465	0	15	47	15	7	84
Taipei City	0.078	374	74	5	2	1	456
Kaohsiung City	0.219	337	262	102	111	79	891
New Taipei City	0.130	601	254	115	45	17	1032
Taichung City	0.658	4	94	188	201	138	625
Tainan City	0.944	0	32	175	236	309	752
Taoyuan City	0.541	20	114	130	129	102	495
**Total**		**1543**	**1539**	**1523**	**1538**	**1535**	**7681**
**%**		**20.09%**	**20.04%**	**19.87%**	**20.02%**	**19.98%**	**100.00%**

**Table 8:** Measures of spatial inequality of community care resources using methods M4–M6 (estimated by 1000*capacity/people).

Method	Median-Mean	Max-Min	Gini Coefficient
M4 Accessibility of nearest simple distance-decay accounting for population of villages and supplier capacity			
	−0.977	124.671	0.497
M5 Accessibility of nearest simple distance-decay accounting for population of villages and supplier loading			
	−0.359	23.228	0.558
M6 Accessibility of nearest moderate distance-decay accounting for population of villages and supplier loading			
	−0.365	24.108	0.562

**Table 9:** Summary statistics of community care resources accessibility measures using methods M3–M6.

Administrative District	Number of Centers										Estimated by 1000*Capacity/People		
		Method M3			Method M4			Method M5			Method M6		
		Mean	Median	SD	Mean	Median	SD	Mean	Median	SD	Mean	Median	SD
Yilan County	80	1.323	0	2.599	5.380	3.676	6.369	1.171	0.867	1.110	1.139	0.812 *	1.141
Hsinchu County	39	0.764	0	1.859	4.419	3.559	3.228	0.673	0.400	0.696	0.654	0.366 *	0.729
Miaoli County	85	1.041	0	1.941	4.443	3.774	3.013	1.105	0.790	1.112	1.083	0.796 *	1.150
Changhua County	112	0.662	0	1.522	3.851	3.534	1.746	0.647	0.435	0.700	0.647	0.429 *	0.701
Nantou County	88	1.155	0	2.462	5.188	4.149	4.682	1.156	0.926	1.046	1.117	0.727 *	1.131
Yunlin County	50	0.417	0	1.171	3.966	3.623	1.894	0.440	0.309	0.363	0.438	0.301 *	0.371
Chiayi County	55	0.652	0	2.108	5.396	4.405	4.113	0.683	0.466	0.928	0.653	0.396 *	0.997
Pingtung County	227	2.419	0	4.179	6.086	4.184	7.571	2.171	1.475	2.157	2.129	1.259 *	2.207
Taitung County	52	2.738	0	5.212	7.394	5.026	7.116	2.546	1.237	3.745	2.488	1.081 *	3.862
Hualien County	33	0.906	0	2.402	6.333	4.608	9.820	0.782	0.508	0.858	0.734	0.434 *	0.950
Keelung City	65	1.332	0	2.012	3.868	3.300	2.394	1.292	0.933	1.287	1.282	0.933	1.293
Hsinchu City	26	0.521	0	1.174	4.500	2.802	11.248	0.573	0.456	0.696	0.571	0.447 *	0.697
Chiayi City	22	0.653	0	1.533	2.566	2.387	1.088	0.683	0.465	0.917	0.682	0.465	0.917
Taipei City	41	0.105	0	0.380	1.242	1.093	0.698	0.100	0.078	0.121	0.099	0.078	0.120
Kaohsiung City	138	0.466	0	1.445	3.723	2.674	5.557	0.480	0.210	0.864	0.468	0.219	0.887
New Taipei City	95	0.239	0	0.998	3.217	2.203	3.162	0.230	0.135	0.241	0.227	0.130 *	0.260
Taichung City	255	0.902	0	1.360	2.655	2.208	1.532	0.853	0.641	0.711	0.850	0.658	0.720
Tainan City	304	1.467	0	2.338	3.961	3.231	2.621	1.431	0.948	1.612	1.427	0.944 *	1.623
Taoyuan City	174	0.850	0	1.326	2.722	2.304	1.800	0.796	0.574	0.687	0.790	0.541 *	0.693
**Average**	**102**	**0.849**	**0**	**2.108**	**3.892**	**2.915**	**4.474**	**0.824**	**0.465**	**1.244**	**0.811**	0.446 *	**1.265**

* (median by M6) − (median by M5) < 0; SD: standard deviation.

**Table 10:** Measures of spatial inequality of community care resources using methods M4–M6.

Administrative District	Method M4		Method M5		Method M6		Composite Index of Inequality (Estimated by Method M6)
	Median-Mean	Gini Coefficient	Median-Mean	Gini Coefficient	Median-Mean	Gini Coefficient	
Yilan County	−1.703 *	0.644 **	−0.304	0.414 *	−0.327	0.411 *	1
Hsinchu County	−0.860	0.635 **	−0.273	0.538 *	−0.289	0.554 *	1
Miaoli County	−0.669	0.564 *	−0.315	0.475 *	−0.287	0.484 *	1
Changhua County	−0.318	0.465 *	−0.212	0.499 *	−0.218	0.503 *	1
Nantou County	−1.039 *	0.665 **	−0.230	0.460 *	−0.390 *	0.478 *	2
Yunlin County	−0.343	0.473 *	−0.131	0.409 *	−0.136	0.421 *	1
Chiayi County	−0.990 *	0.569 *	−0.216	0.515 *	−0.257	0.517 *	1
Pingtung County	−1.902 *	0.687 **	−0.696	0.592 *	−0.869 *	0.585 *	2
Taitung County	−2.368 *	0.701 **	−1.309 *	0.735 **	−1.406 *	0.740 **	3
Hualien County	−1.725 *	0.706 **	−0.274	0.568 *	−0.300	0.589 *	1
Keelung City	−0.568	0.543 *	−0.359	0.484 *	−0.349	0.479 *	1
Hsinchu City	−1.698 *	0.695 **	−0.117	0.454 *	−0.124	0.455 *	1
Chiayi City	−0.180	0.369	−0.218	0.427 *	−0.217	0.425 *	1
Taipei City	−0.149	0.402 *	−0.022	0.412 *	−0.020	0.410 *	1
Kaohsiung City	−1.049	0.645 **	−0.270	0.661 **	−0.248	0.653 **	2
New Taipei City	−1.015	0.618 **	−0.095	0.527 *	−0.096	0.528 *	1
Taichung City	−0.448	0.533 *	−0.212	0.445 *	−0.192	0.446 *	1
Tainan City	−0.730	0.587 *	−0.483 *	0.573 *	−0.483 *	0.574 *	2
Taoyuan City	−0.418	0.486 *	−0.223	0.472 *	−0.249	0.473 *	1
**Average**	**−0.977**	0.497 *	**−0.359**	0.558 *	**−0.365**	0.562 *	

*“median-mean” is smaller than “Average”; * Gini coefficient is between 0.4~0.6; ** Gini coefficient is above 0.6.
